# Massive Pulmonary Embolism Associated With Factor V Leiden Mutation in a Young Female on Oral Contraceptive Pills: A Case Report

**DOI:** 10.7759/cureus.62451

**Published:** 2024-06-15

**Authors:** Sushil Rayamajhi, Gabriela Sayonara Lopez Capa, Ligia Carolina Flores Reyes, Vyshnavidevi Sunkara, Tania Beatriz Marin Padilla, Ameer M Farrukh, Anil Harrison

**Affiliations:** 1 Internal Medicine, University of Central Florida/Hospital Corporation of America, Florida West Hospital, Pensacola, USA; 2 Medicine, Universidad Católica de Cuenca, Azuay, ECU; 3 Medicine, Universidad Nacional Autónoma de Honduras, Tegucigalpa, HND; 4 Internal Medicine, Katuri Medical College, Guntur, IND; 5 Medicine, University of Galway, Galway, IRL; 6 Internal Medicine, Florida State University, Pensacola, USA; 7 Internal Medicine, University of Central Florida, Pensacola, USA

**Keywords:** venous thromboembolism (vte), venous thromboembolism, pulmonary embolism, ocp, factor v leiden

## Abstract

Factor V Leiden (FVL) is the major genetic risk factor to predispose venous thromboembolism (VTE). We present a rare case of a 34-year-old Caucasian female heterozygous for this mutation and taking oral contraceptive pills (OCPs) for less than four months, who presented to the emergency department with acute onset of dyspnea and was diagnosed to have an isolated massive bilateral pulmonary embolism (PE). The patient was managed for six days in the hospital and was discharged on oral anticoagulants. The risk for VTE in patients with factor V Leiden and on oral contraceptive pills increases by 30-fold in heterozygous women and 100-fold in homozygous women. The risk of VTE in factor V Leiden seems to outweigh the benefit of contraception with oral contraceptive pills. This case suggests that thrombophilia screening should be considered only in patients with a positive first-degree family history of VTE, where necessary, to prevent any future thrombotic morbidity and mortality.

## Introduction

Venous thromboembolism (VTE) encompasses both deep vein thrombosis (DVT) and pulmonary embolism (PE) [1]. VTE at an early age is mostly associated with the presence of inherited disorders. The most common genetic risk factor for PE in Caucasians is the factor V Leiden (FVL) mutation, with a prevalence of 15%-25%. FVL is due to a single nucleotide base substitution of arginine with glutamine, which occurs at amino acid 506 of the factor V gene. This renders activated protein C (APC) unable to cleave Va and VIIIa. Thus, this inhibits and delays clotting [2]. Additional risk factors also increase the risk of VTE, especially malignancy, surgery, trauma, oral contraceptive pills (OCPs), hormonal replacement therapy, pregnancy, and immobility. Diagnosis involves determining the pretest probability with a Wells Score, D-dimer testing, and imaging. A computed tomography (CT) scan can be used to detect a PE [3]. Treatment for PE involves using anticoagulation therapy such as low-molecular-weight heparin (LMWH), unfractionated heparin (UFH), or direct oral anticoagulants (DOACs) [4].

## Case presentation

A 34-year-old Caucasian female presented to the emergency department with acute sudden onset of moderate sharp pleuritic chest pain, shortness of breath, and chest tightness for one day, which worsened with exertion. She had a past medical history of factor V Leiden (diagnosed at age 20) and a positive family history of factor V Leiden and deep vein thrombosis (DVT) in her father and grandfather. The patient denied a previous history of VTE, recent surgery, extended periods of immobility, anticoagulant use, smoking, or cancer. On further history taking, she admitted being on oral contraceptive pills for four months duration.

On general assessment, the patient was alert, oriented, and breathing shallow due to sharp chest pain on deep inspiration. Her heart rate was 90 beats/minute, and SpO2 improved on 2 L nasal cannula to 96%. Lung examination revealed bilateral vesicular breath sounds without wheezing or crepitation. The physical examination revealed the absence of leg edema or calf tenderness. Laboratory results including complete blood count (CBC), troponin, arterial blood gas (ABG), serum chemistry, and toxicology screening were within normal range. Electrocardiogram (EKG) revealed sinus rate and rhythm, and T wave inversion in lead III and lead V3. Echocardiogram showed a normal ejection fraction of 55% with the absence of a right ventricular strain pattern. Her D-dimer level was elevated at 1,560 ng/mL (normal limit: 0-500 ng/mL). Bilateral lower extremity venous Doppler showed no evidence of deep vein thrombosis.

**Figure 1:**
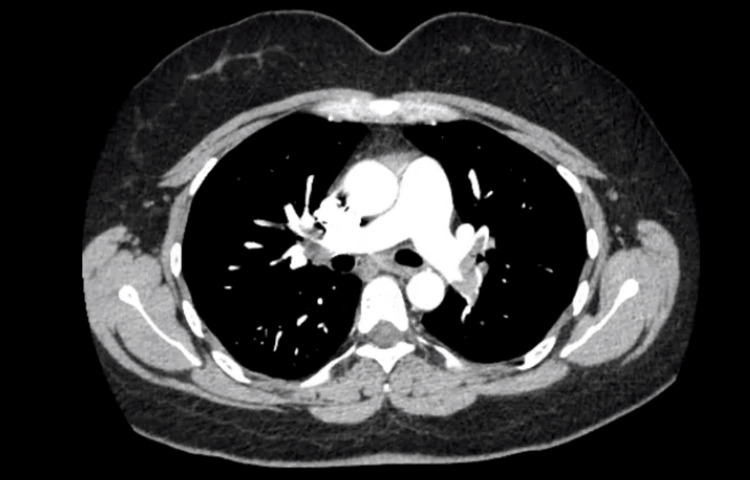
CT angiogram showed large bilateral central pulmonary arteries extending into the lobar and segmental vessels bilaterally with no evidence of right heart strain CT: computed tomography

Due to high suspicion of PE, a computed tomography pulmonary angiogram (CTPA) was done, which revealed large bilateral central pulmonary arteries extending into the lobar and segmental vessels bilaterally with no evidence of right heart strain and no pleural effusion (Figure 1).

The echocardiogram excluded the presence of right heart strain without enlargement of the right ventricle. Given the exclusion of right heart strain, PE was considered mild-risk PE, and a thrombectomy procedure was not required.

She was started on unfractionated heparin (UFH) and later switched to apixaban (Eliquis) on discharge for six months. She was hemodynamically stable during her hospital stay. Her chest pain persisted for a couple of days and ultimately resolved with analgesia. The patient's clinical condition improved, and she was discharged on the fourth day, to follow up as an outpatient. She was advised about the avoidance of risk factors and to follow up with hematology and gynecology. Follow-up at two weeks and six months showed significant clinical improvement.

## Discussion

Venous thromboembolism can clinically manifest either as deep vein thrombosis or pulmonary embolism [1]. In the United States, VTE ranks as the third leading cause of cardiovascular death [2]. Symptoms of pulmonary embolism are nonspecific, including dyspnea, chest pain, presyncope/syncope, and hemoptysis. In rare circumstances, patients may exhibit hemodynamic instability indicating central or extensive PE [5]. The causes of pulmonary embolism are diverse, including both environmental and genetic. Genetic predisposition plays a significant role in VTE, with approximately 50%-60% of the variability attributed to genetic factors [6]. Genetic risk factors include antithrombin deficiency, protein C deficiency, protein S deficiency, factor V Leiden mutation, and factor II 202lOA mutation [7]. Factor V Leiden, the most prevalent genetic risk factor, is present in 20%-25% of patients with VTE and 50% of patients with familial thrombophilia [8,9]. Environmental risk factors contributing to VTE include malignancy, trauma, immobilization, pregnancy, hospitalization, long-haul travel, age, and oral contraceptive pill usage.

Factor V Leiden (FVL) has an autosomal dominant inheritance pattern and demonstrates incomplete penetration. This indicates that not every person with the mutation will develop the condition. This mutation, resulting from guanine to adenine substitution in the factor V gene, impedes activated protein C (APC) from cleaving factor Va and VIIIa, thereby prolonging plasma clotting time [10]. Factor V Leiden significantly elevates the risk of VTE, with a 5% annual risk rate. A cost-efficient method for detecting factor V Leiden (FVL) involves assessing APC resistance using a second-generation coagulation assay. This testing approach demonstrates 100% sensitivity and specificity for identifying FVL [11]. Indications for thrombophilia screening include VTE associated with hormonal contraception, a first unprovoked VTE at any age, and a first VTE with a first-degree family history of VTE before age 50 [11]. Oral contraceptive pills are a well-established risk factor for VTE. Several studies have found that the risk increases with increasing estrogen dose [12,13]. In women with factor V Leiden, the risk of VTE significantly rises by 30-fold in heterozygous individuals and 100-fold in homozygous individuals when combined with oral contraceptive pills [9]. Testing for thrombophilia conditions in patients diagnosed with the first episode of DVT/PE is being discouraged according to the American Society of Hematology (ASH) guidelines as it is expensive with no clinically meaningful benefit.

Computed tomography pulmonary angiogram (CTPA) is the preferred imaging modality of choice for PE, offering detailed visualization down to the segmental level of the vascular system [5]. Acute PE can lead to pressure overload and dysfunction of the right ventricle, which can be detected by an echocardiogram. It has a negative predictive value of 40%-50%, and thus, negative findings cannot exclude PE. In most cases, a PE originates from a DVT in the lower limb. In a study that involved venography, DVT was observed in 70% of patients with PE [14]. In our case, bilateral Doppler venography showed no evidence of DVT in bilateral lower limbs.

Management for PE consists of three components: cardiopulmonary support, anticoagulation to prevent worsening or recurrence, and pulmonary vasculature reperfusion. Low-molecular-weight heparin (LMWH) is preferred over unfractionated heparin due to its ability to reduce thrombus size and complications. According to the therapeutic guidelines for acute PE, a recommended dosage is enoxaparin at 1.5 mg/kg daily, with dose adjustment necessary if creatinine clearance is <30 mL/minute. Alternatively, warfarin therapy can be initiated, maintaining an international normalized ratio (INR) between 2 and 3. Direct oral anticoagulants, such as apixaban or rivaroxaban, are also currently approved for PE management. The duration of treatment is usually six months but may vary based on risk factors [15]. In cases of massive PE, patients may undergo pulmonary artery reperfusion therapy, such as systemic or catheter-directed thrombolysis using tissue plasminogen activator (tPA). Systemic thrombolysis has been shown to decrease mortality rates to 2.4% in cases of massive PE. However, risks include bleeding, and thus, contraindications of thrombolysis should be considered [15].

## Conclusions

The management of pulmonary embolism (PE) is most effective when approached with a multidisciplinary strategy. All the potential risk factors must be considered when evaluating a patient with PE. In particular, young patients with a significant family history of venous thromboembolism (VTE) should be evaluated for thrombophilia through genetic screening. This case report demonstrates the heightened risk of venous thrombosis in females with heterozygous factor V Leiden who take oral contraceptive pills (OCPs), emphasizing the importance of identifying such individuals before OCP initiation. Routine screening for factor V Leiden would be time-consuming and costly and therefore is a medical decision. Timely diagnosis, patient education, lifestyle modifications, and appropriate anticoagulation therapy are essential components of effective PE management.
